# Texture changes during chilled storage of wild and farmed blackspot seabream (*Pagellus bogaraveo*) fed different diets

**DOI:** 10.1002/fsn3.2516

**Published:** 2021-09-14

**Authors:** Pedro Luis Castro, Laura Rincón, Blanca Álvarez, Rafael Ginés

**Affiliations:** ^1^ GIA‐ECOAQUA Universidad de Las Palmas de Gran Canaria Telde, Las Palmas Spain; ^2^ IEO Centro Oceanográfico Vigo Spain

**Keywords:** blackspot seabream, chilling, histology, quality, texture, wild and cultivated

## Abstract

The impact of changes in dietary lipids and protein sources on texture was evaluated on farmed blackspot seabream (*Pagellus bogaraveo*) throughout 14 days of ice storage and compared with wild fish. A commercial diet formulated with a high proportion of lipids, and two diets formulated with an important reduction of lipid levels by 60% and adding either plant protein sources (LL diet) or fishmeal (LL + diet) were supplied during growth until commercial size was attained. In the wild fish, the raw fillet hardness was significantly higher than in farmed fish during the entire ice‐storage period. In the farmed fish, an increase of muscle lipid accumulation and change of fiber density were responsible for the variations in texture in the raw fillet. The highest reduction was found in fish fed with diets LL+ and LL. The texture parameters studied on the cooked fillets showed no significant differences, neither attributable to the diets nor to the ice‐storage period.

## Graphical abstract

The article examines the effect on fillet texture of a reduction of dietary lipids for farmed blackspot seabream, a promising marine fish species for farming purposes. According to the present results, it is possible an important fish oil reduction in the commercial diets used in blackspot seabream with a slight variation in texture.

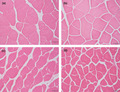

## 1 INTRODUCTION

The blackspot seabream (*Pagellus bogaraveo*) is a fish species with an elevated commercial value, thanks to its flesh firmness and flavor. The aquaculture production of blackspot seabream has become a promising market alternative considering that this semi‐pelagic marine fish, commonly found off the European coasts, is being overfished and causing the general collapse of traditional fisheries (Lorance, 2011; Pinho et al., 2014). Hence, captures have decreased from 8,910 tons in 1980 to 1,385 tons in 2018 (FAO, 2021). Concerning diet ingredients, the global demand for fishmeal and fish oil has steadily increased the costs of the diets for farmed fish (Tacon and Metian, 2008; Castro et al., 2015). In this context, blackspot seabream represents an interesting opportunity, as carnivorous species maintain a reasonably good growth potential despite the reduction in the inclusion of fish oil in practical diets (Figueiredo‐Silva et al., 2009). This desirable performance is conditioned upon an enhancement of energy available via the inclusion of carbohydrates, only effective in maintaining growth parameters while the protein source comes from fishmeal (Valente et al., 2010).

Fish farming promotes compositional variations that affect flesh quality (Izquierdo et al., 2003). Diet ingredients and a constant supply of food result in large deposits of lipids, as previously reported on different farmed species, such as the gilthead seabream (*Sparus aurata*) (Grigorakis et al., 2002), European seabass (*Dicentrarchux labrax*) (Alasalvar et al., 2002; Fuentes et al., 2010) or Atlantic halibut (*Hippoglossus hippoglossus*) (Olsson et al., 2003), especially as compared with their wild counterparts. Research on blackspot seabream has focused on fatty acid composition (Álvarez et al., 2009; Laconisi et al., 2017). Concerning quality, Sant’Ana et al. (2011) developed a QIM (Quality Index Method) specific for the species. Comparing wild and farmed blackspot seabream, Rincón et al. (2016) showed differences in fat proportion and collagen composition. In addition, a shelf life assessment was made, comparing wild and farmed blackspot seabream (Castro et al., 2018), based on fatty acid profiles, pH, muscle color, and QIM.

In the field quality, texture is one of the most important parameters, not only for producers but also for consumers (Hyldig and Nielsen, 2001), since the firmness, changes throughout the shelf life are closely associated with acceptability (Cheng et al., 2014). In farmed fish, the ample lipid content and its distribution influence the texture properties of the flesh (Lie, 2001) that can be modulates through the formulation of diets. Suárez et al. (2010) reported, after a feed restriction, an enhancement in the muscle texture associated with the decrease of lipid deposits in the structural components of the muscle. The muscle structure of farmed fish has been associated with a softer texture than that of wild fish (Periago et al., 2005). Factors of potential relevance in fish farming, such as diet composition and feeding regimes, promote variations during muscle growth in the muscle fibers, as distribution or girth (García de la Serrana et al., 2013). The effect is noticeable in the two main processes: the hyperplastic input of new fibers, known to occur in the skeletal muscle of blackspot seabream (Silva et al., 2009), and the hypertrophic growth of previously formed fibers (Alami‐Durante et al., 2010), resulting from the balance between protein synthesis and degradation. Accordingly, histological studies of fiber recruitment, morphology, or distribution are a suitable instrument to understand texture properties of the species under study.

The present work focuses on those changes in the density of the muscular fibers and those texture variations on farmed blackspot seabream fed different diets and compared with their wild counterparts, during chilled storage.

## 2 MATERIALS AND METHODS

### 2.1 Growth trial

FELASA category C endorsements and the European Economic Community animal experimentation guidelines directive of 24 November 1986 (86/609/EEC) and (2010/63/EU) were followed. Blackspot seabream (mean weight of 155.1 ± 30.4 g, 14 months old) from the Oceanographic Spanish Institute (Vigo, Spain), were fed 20 months three diets, Control, LL, and LL+. These diets aimed to drastically reduce the lipid content of the commercial diet (Control) commonly used for the species, similar to that developed for other Sparidae such as gilthead seabream. However, black spot seabream is leaner than gilthead seabream and maintain adequate production yields with a lower lipid ratio. The energy balance to compensate with carbohydrates here is regulated by the quality of the protein in the diet. Thus, the Control diet (Bes‐Power, Sparos, Faro, Portugal) consist of lipids (29.33% dry weight), mostly from fish oil (170 g/kg), and two thirds from plant sources and one third from fishmeal as protein content. To decrease lipid levels by 60% compensating the energy intake by including carbohydrates, two experimental diets were formulated, LL (low lipid diet) and LL+ (low lipid diet +fish meal). In LL, fish oil reduced (50 g/kg), but the proportions of protein sources were maintained. In LL+, fish oil reduction did not vary (50 g/kg), but most of the protein come from fishmeal (456 g/kg in LL+diet against 216 g/kg in LL diet).

### 2.2 Sample preparation

At the end of the experimental trial, 30 farmed blackspot seabream per diet (405.2 ± 68.6.4 g), were sampled. Furthermore, 30 wild fish (509.0 ± 46.8 g), were obtained upon arrival at Vigo Fish Market (Pontevedra, Spain) within 10 hr of being caught. Fish were packed as whole ungutted fish with flaked ice into polystyrene boxes with holes for drainage ice and shipped to the High Specialization Aquaculture and Biotechnology Service (SABE) (ULPGC), arriving within 24 hr. Fish were stored at 4℃ for 14 days postharvest (dph). During storage, five fish per diet, randomly chosen, were obtained at 1, 4, 7, 10, and 14 postharvest day (dph) and individually sampled.

### 2.3 Texture profile analysis

The Texture Profile Analysis (TPA) was made using a TA.XT2 Texture Analyzer (Stable Micro Systems Ltd.). The analysis comprises whole ungutted fish and raw and cooked fillets carried out on six different fish from each dietary group. For whole fish, the compression was made over the lateral line at one cm from the operculum. Two successive cycles with a plunger of 12 mm Ø to mimic the compression applied by a researcher during a sense evaluation (Ginés et al., 2002). Depth 7 mm, speed 0.8 mm/s (5 s between cycles). The left fillet was unskinned and then divided in square pieces (2.5 × 2.5 × 1.5 cm) collected above the lateral line from cranial, central, and caudal location. For fillet texture examination, a compression plate (100 mm Ø) at 0.8 mm/s were used, forcing a deformation (60% of the original thickness) (Ginés et al., 2004). Following the same procedure that for raw fillet, three fragments were baked in an air‐heated oven (Compact Eurofred, Barcelona, Spain) at 115℃, for 10 min in packed in aluminum boxes. The deformation of the original length for cooked fillet was 80%.

### 2.4 Proximate composition

Fish fillets from the right side were homogenized in batches of three and immediately and subjected to proximal analysis by a FoodScan™ (FOSS) based on NIR transmittance technology for the simultaneous determination of moisture, protein, and fat content in meat and meat products (Anderson et al., 2007). Dry matter content was calculated by drying in an oven (110ºC) until constant weight, ash content by combustion in a muffle furnace (600ºC for 12 hr, AOAC, 1995).

### 2.5 Histology. Muscle fiber studies

At the end of the experiment, 15 fish per diet were sampled. Muscle tissue from the medial section, under the lateral line, was fixed in 10% neutral‐buffered formalin, dehydrated in an ethanol series and embedded in paraffin wax. Sections of five µm were prepared with a Leica microtome (Leica Instruments GmbH) and stained with hematoxylin and eosin (Luna, 1968) for histological evaluation. Fiber number among groups was evaluated with an image analysis package (Image‐Pro Plus software, Media Cybernetics) attached to a photomicroscope (Olympus CX41). Three different microphotographs were randomly taken per section (10× objective magnification). To determine the fiber density, three measurements, at separated positions of each image (nine per fish), were recorded and subsequently averaged. Fiber density (fibers mm^−2^) was calculated as the number of fibers per mm^2^ of muscle cross‐sectional area (Rincón et al., 2016).

### 2.6 Statistical analysis

Data were submitted to a general linear model with diet and time of storage as fixed factors and body weight as a covariate using a SPSS Statistical Software System 26.0 (Armonk, NY: IBM Corp.). Those significant differences were evaluated by Duncan's multiple range tests. Pearson's correlation analysis determined those interactions between biochemical composition and texture parameters.

## 3 RESULTS AND DISCUSSION

The total fat content was twofold lower in the wild blackspot seabream than in the farmed fish (Table 1), thus showing that farmed fish can increase the proportion of muscle lipid when fed high‐energy diets. Studies on blackspot seabream (Silva et al., 2006) have referred to the protein/lipid ratio as a source of variation in the fat content of the whole body. Figueiredo‐Silva et al. (2010), in order to study the effect of protein source, formulated some experimental diets with different proportions of fish meal and vegetable ingredients. These inclusions produced, in juvenile fish, a significant impact on the fat content. However, at the commercial size, as in the present study, no differences based on the protein/lipid ratio or the protein sources were recorded. This means that the metabolism of full growth fish from lean species, like blackspot seabream, shows a poor utilization of dietary lipids (Valente et al., 2011), accumulating similar fat amounts in the muscle independently of the fed diets either from fish or vegetable sources. The deposition of lipids could increase, though, from dietary protein when the ratio protein/energy increases (Francis and Turchini, 2017) as in the low‐lipid diets LL and LL+.

**TABLE 1:** Proximal composition (g/100 g wet muscle) of the muscle of wild blackspot seabream and blackspot seabream fed different diets (Mean ± *SD*)

	Diet			
	Control	LL	LL+	Wild
Protein	20.48 ± 0.40^a^	19.77 ± 0.35^b^	20.55 ± 0.46^a^	18.99 ± 0.67^c^
Lipid	2.99 ± 0.09^a^	3.96 ± 0.81^a^	3.60 ± 0.77^a^	0.91 ± 0.09^b^
Moisture	74.85 ± 0.40^b^	73.21 ± 0.79^c^	75.52 ± 0.96^b^	78.57 ± 0.76^a^
Ash	2.03 ± 0.35^a^	1.42 ± 0.04^b^	1.44 ± 0.04^b^	1.96 ± 0.24^a^

Note

Different letters in the same line denote statistically significant differences (*p* < .05).

The influence of fat content in the muscle on the texture of the fillet is shown in Table 2. Texture studies on different fish species have reported a significant loss of hardness, and hence a softening of the flesh associated with the increment of the fat content (Ginés et al., 2004; Ingebrigtsen et al., 2014; Másílko et al., 2015; Menoyo et al., 2004; Thakur et al., 2003, 2009). This relationship associated with a fattier flesh, even when it is not systematically observed, always leads to a softer texture (Lefevre et al., 2015). Thus, studying the hardness in whole blackspot seabream, or in the fillet, raw or cooked (Table 2), the muscle fat content was negatively correlated with the maximum force to compression. The highest influence of fat content was recorded in the raw fillet of the blackspot seabream, explaining more than 30% of the total variation. This value is around threefold higher than that found by Aussanasuwannakul et al. (2011) for rainbow trout (*Oncorhynchus mykiss*), probably based on the ample range of fat content deposited in the muscle of lean species, as blackspot seabream, when comparing farmed with wild fish. In contrast, studying Atlantic salmon (*Salmo salar*) a representative oily fish, Johnston et al. (2006) found no correlation between lipid content and fillet hardness. Despite the significant increase of lipid content from 46% to 84%, comparing wild and farmed salmon, these lipids in the muscle were not able to explain the observed differences in texture. When the farmed fish is fed diets that promote important differences in fat muscle storage, a significant negative correlation between muscle lipid content and flesh hardness could be explained for 50% of the variation (Xu et al., 2016). In our case, the fat increase was three or four‐fold larger and similar to that reported by Fuentes et al. (2010), comparing wild and farmed European seabass. The total fat content in the muscle was positively correlated in both raw and cooked fillet, studying shape recovery after the first compression, the springiness (Table 2). However, total protein content had a significant role, especially in the raw fillet, being more important than total fat content to determine the springiness and it explained, together with fat content, around a 40% of the total recorded variation. Regarding proximate parameters throughout chilling time can vary depending upon a range of factors such as species, physiological condition, stress before slaughter, and storage temperature (Castro et al., 2010, 2018).

**TABLE 2:** Pearson's correlation coefficients between chemical composition and texture parameters (hardness and springiness) of whole fish, raw fillet and cooked fillet (Pearson's coefficient and *p* value) (*n* = 200)

	protein	lipid	whole hard	whole spring	raw hard	raw spring	cooked hard
lipid	.204						
	*.021*						
whole fish hardness	.219	‐.352					
	*.014*	*.000*					
whole fish springiness	.346	.094	.258				
	*.000*	*.176*	*.005*				
raw fillet hardness	.014	‐.579	.565	.285			
	*.444*	*.000*	*.000*	*.002*			
raw fillet springiness	.540	.297	.091	.216	‐.208		
	*.000*	*.001*	*.183*	*.016*	*.019*		
cooked fillet hardness	.031	‐.259	.152	‐.005	.247	.003	
	*.379*	*.005*	*.065*	*.482*	*.007*	*.489*	
cooked fillet springiness	.271	.323	‐.335	.026	‐.363	.062	‐.318
	*.003*	*.001*	*.000*	*.400*	*.000*	*.271*	*.001*

The values of hardness registered on whole fish were positively correlated with those of raw fillet, despite the effect of skin integrity on the maintenance of muscle structure and the difficulty to assess the texture of whole fish due to the lack of a uniform structure (Hyldig & Nielsen, 2001). Correlation statistics between hardness of raw and cooked fillets were positive and significant but low (Table 2). It has been described that after cooking, the effect of muscle fat content on the mechanical resistance of raw flesh is no longer observed (Lefevre et al., 2015).

**FIGURE 1:**
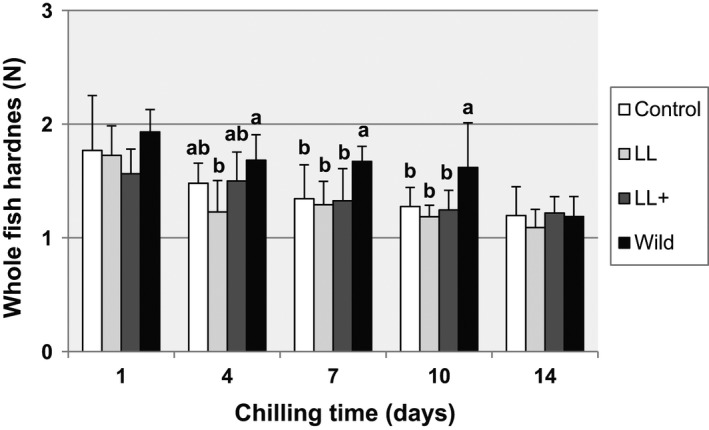
Whole fish hardness (N) of blackspot seabream, wild, and fed different diets, throughout ice storage. Different letters in the same day denote statistically significant differences *n* = 30 (*p* < .05)

The evolution of the texture parameters throughout shelf life varied, depending on the experimental diets. Whole fish hardness showed no differences on 1 dph, but some differences appeared from 4–10 dph, with the highest for wild fish. At the end of the ice‐storage period, on the 14 dph, the maximum compression force studied on whole fish was not affected by the dietary treatments (Figure 1). We found no differences from 4–14 dph between each treatment except in diet LL, which was significantly lower on 14 dph, than the other storage days of this diet. A tendency for springiness to diminish can increase during ice storage in all treatments, although differences were only significant between 1–14 dph (data not shown).

**FIGURE 2:**
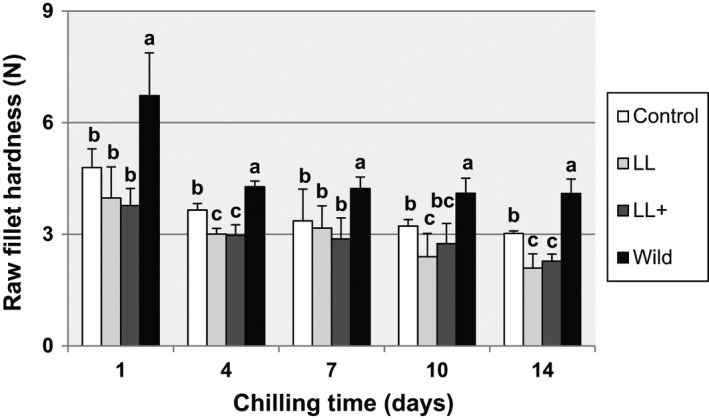
Raw fillet hardness (N) of blackspot seabream, wild, and fed different diets, throughout ice storage. Different letters in the same day denote statistically significant differences *n* = 30 (*p* < .05)

The raw fillet hardness was significantly higher in wild fish than fish‐fed experimental diets during the entire chilling time (Figure 2), with differences especially remarkable on 1 dph. All groups showed a significant decrease in values of raw fillet hardness, comparing 1 dph with the other days of sampling. However, the wild fish did not show differences between 4–14 dph, while in the other treatments the reduction of the values obtained was significant. The greatest reduction was found in fish fed the LL +and LL diets.

Thus, the replacement of fish oil in the diets did not affect instrumental texture parameters when the fat content in muscle was not varied, as previously described in gilthead seabream (Matos et al., 2012). On the other hand, the springiness of raw fillet was lower in wild fish but only significantly different from 4–10 dph (data are not shown). There were no differences between the other treatments on each dph, or between days of treatment, except 1 and 14 dph in fish fed diet LL+.

No significant differences in texture parameters were found studying the cooked fillets, neither attributable to the treatment nor the period of ice storage. Only the springiness was statistically lower in wild fish at 1 dph compared with the other treatments (data not shown). After cooking, the collagen shrinks then softens, whereas the actomyosin complex changes from a soft gel to a firmer denatured complex, making it very difficult to relate the texture attributes of raw flesh to the attributes once the fillet is heated (Hyldig and Nielsen, 2001).

**FIGURE 3:**
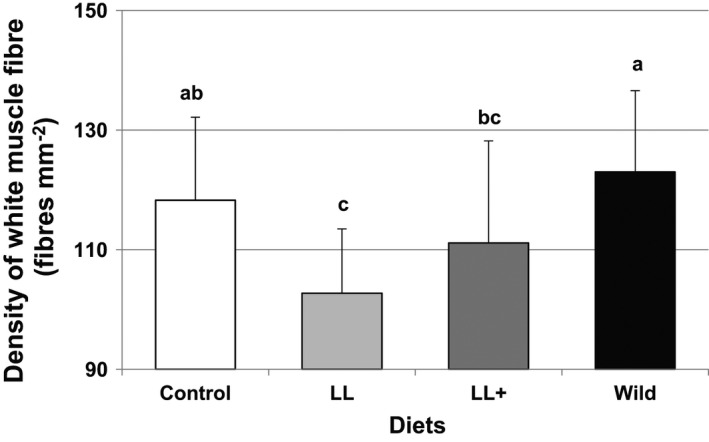
Density of white muscle fibers (fibers mm^−2^) of blackspot seabream, wild, and fed different diets. Different letters in the same day denote statistically significant differences *n* = 30 (*p* < .05)

**FIGURE 4:**
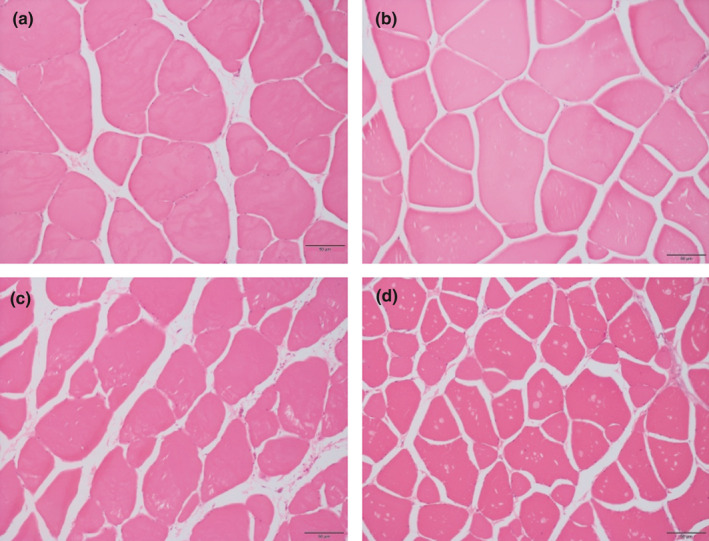
White fibers amount of the blackspot seabream fillet. Hematoxylin–eosin 20×. (a) LL diet. Farmed fish, white muscle. (b) LL+diet. Farmed fish, white muscle. (c) Control diet. Farmed fish, white muscle. (d) Wild fish, white muscle

The images of white muscle sections from the blackspot seabream fed experimental diets are shown in Figure 3. Morphologically, they are square shaped fibers, with no uniform size, including small diameter fibers intermingled with larger fibers, giving the muscle a characteristic mosaic appearance that results from the hyperplasia process (Castro et al., 2015; Johnston, 2001). Previous studies of blackspot seabream have outlined the muscle growth kinetics, emphasizing hyperplasia as the main relative contributor to the increase of white muscle from larvae to juvenile size (Silva et al., 2008). In wild fish, the density of white muscle fibers was higher than that in the farmed fish, but only significantly different as compared with the muscle of fish fed diets with low‐lipid content, LL and LL+. Among the farmed fish, those fed the control diet computed the highest number of fibers, significantly superior to those from fish fed diet LL (Figure 4). The changes in the muscle of the fish over the chilling time have a significant impact on fillet quality and consumer acceptance. This evolution of the fiber morphology does not affect the proportion of fibers. Tissue degradation is accompanied by myofibrillar proteolysis produced as a consequence of the activation of proteolytic enzymes (Caballero et al., 2009). The progressive detachment between myofibers and the myocommata conditioned the reduction in flesh hardness as observed in the present study.

Muscle cellularity is considered a determining factor for assessing texture characteristics (Johnston, 1999; Palstra and Planas, 2011). These studies have pointed out that the density of the muscle fibers holds a positive and significant correlation with texture parameters. High fiber density represents a larger surface‐to‐volume ratio, and so the connective tissue surrounding each fiber would be relatively more abundant than in a muscle with low fiber density (Periago et al., 2005). In the present study, the wild blackspot seabream, with a large fiber density (Figure 4), showed the highest values of hardness and springiness studied on the raw fillet (Figure 2). This fact does not apply to the cooked fillet, since the correlations with the mechanical resistance parameters are less relevant (Lefevre et al., 2015) as discussed below. Studying wild European seabass, Periago et al. (2005) found a higher muscle density than in the farmed specimens, and in agreement with the present study, the muscle of wild fish showed the highest values of texture parameters. Similarly, Johnston et al. (2006) reported that wild Atlantic salmon had a firmer texture than that in the farmed fish. However, the authors concluded that with the shear test that was used in their experience, the reported firmness was related to the amount of insoluble hydroxyproline, more than to differences in muscle cellularity. The shear test applies only one deformation to the sample and thus gives no measure of how much of the applied work is absorbed as elastic deformation (Veland and Torrissen, 1999), depending mainly on the muscle fiber disposition.

In farmed fish, the number of muscle fibers and fillet texture are influenced by rearing factors, including exercise (Rasmussen et al., 2013), photoperiod (Johnston et al., 2003) and diet formulation (García de la Serrana et al., 2013). Regarding exercise, the improvement of texture parameters as hardness or springiness would be conditioned by the highest white fiber density associated with water velocity into the tank (Li et al., 2016). Light treatment affected muscle growth over the production cycle in salmon, resulting in a large fiber density and a firmer flesh, when continuous photoperiod was applied (Johnston et al., 2003). Finally, and related to diet, Alami‐Durante et al. (2010) reported significant changes in the distribution of the girth of the white muscle fibers, depending on the level of fishmeal substitution by different plant protein sources. That effect could explain those variations in the blackspot seabream fed the experimental diets with low lipid content. Thereby, fish fed diet LL showed a lower fiber density than fish fed diet LL+, due to a lower content of fishmeal. In any case, differences were not significant so that they do not influence the texture profile.

Conversely, the comparison of the results from the fish fed the control diet with the low lipid diets, LL and LL+, showed the highest fiber density on the diet with the highest lipid content, the control diet. Variations of soybean meal content could have promoted these differences and their inclusion would lead to a decrease in the mean and median diameters of muscle fibers, as has been related in species such as rainbow trout (Alami‐Durante, Wrutniak‐Cabello, et al., 2010). Hence, it seems that the level and origin of the protein of the diet composition influences the muscle growth dynamics, while the replacement of fish oil with vegetable sources has less impact on fiber size (Haugen et al., 2006; Matos et al., 2012).

Therefore, mutually, the size and lipid content of the muscle fibers contributed to the mechanical resistance of the raw fillet; not only the particular responsibility of adipocytes with less resistance to compression, but also the muscle fibers that are bathed in large amounts of lipid and can slide more easily across each other and generate less resistance (Aussanasuwannakul et al., 2011). The collagenous connective tissue structure can contribute to the structural weakening of the muscle (Thakur et al., 2003). Particularly, the highest proportion of intramuscular adipocytes, in the farmed fish muscle, located within the perimysium and myosepta, resulting in a mechanically less resistant tissue as compared with a lean tissue, rich in fibrous proteins (Lefevre et al., 2015). In cooked fillet, the muscle segments tend to slide upon compression because fish has a flaky structure and during heating, the connective tissue that holds the flakes together dissolves (Castro et al., 2015; Hyldig and Nielsen, 2001). This makes the fish muscle fragile with handling after cooking, and it separates easily into flakes.

## 4 CONCLUSIONS

The reduction of fish oil in the commercial diets destined for the blackspot seabream and its replacement by meals, whether of vegetable or fish origin, promotes variable changes in the texture of the fillet during the marketable period. The density of muscle fibers decreases along with the level of dietary fish oil, a fiber reduction boosted with the inclusion of vegetable meal. The reduction of costs of the diet should be weighed, considering the decline in the texture attributes of the fish, detected only in advanced stages of the marketable period. Further sensory analysis will aid the understanding of minor sensory attributes changes.

## CONFLICT OF INTEREST

The authors declare that they have no conflict of interest.

## AUTHOR CONTRIBUTIONS

**Laura Rincón :** Investigation (supporting). **Blanca Álvarez :** Conceptualization (supporting); Investigation (supporting). **Rafael Ginés :** Conceptualization (lead); Data curation (lead); Formal analysis (supporting); Funding acquisition (lead); Investigation (supporting); Methodology (lead); Project administration (supporting); Resources (equal); Supervision (equal); Validation (equal); Writing‐original draft (equal).

## ETHICAL APPROVAL

All applicable international (Directive 2010/63/EU), national, and/or institutional guidelines for the care and use of animals were followed by the authors.

## Data Availability

The data that support the findings of this study are available on request from the corresponding author. The data are not publicly available due to privacy or ethical restrictions.

## References

[fsn32516-bib-0001] Alami‐Durante, H. , Médale, F. , Cluzeaud, M. , & Kaushik, S. J. (2010). Skeletal muscle growth dynamics and expression of related genes in white and red muscles of rainbow trout fed diets with graded levels of a mixture of plant protein sources as substitutes for fishmeal. Aquaculture, 303, 50–58. 10.1016/j.aquaculture.2010.03.012

[fsn32516-bib-0002] Alami‐Durante, H. , Wrutniak‐Cabello, C. , Kaushik, S. J. , & Médale, F. (2010). Skeletal muscle cellularity and expression of myogenic regulatory factors and myosin heavy chains in rainbow trout (*Oncorhynchus mykiss*): Effects of changes in dietary plant protein sources and amino acid profiles. Comparative Biochemistry and Physiology Part A, 156, 561–568. 10.1016/j.cbpa.2010.04.015 20434580

[fsn32516-bib-0003] Alasalvar, C. , Taylor, K. D. A. , Zubcov, E. , Shahidi, F. , & Alexis, M. (2002). Differentiation of cultured and wild sea bass (*Dicentrarchus labrax*): Total lipids content, fatty acid and trace mineral composition. Food Chemistry, 79(2), 145–150. 10.1016/S0308-8146(02)00122-X

[fsn32516-bib-0004] Álvarez, V. , Trigo, M. , Lois, S. , Fernández, D. , Medina, I. , & Aubourg, S. P. (2009). Comparative lipid composition study in farmed and wild blackspot seabream (Pagellus bogaraveo) (vol. 27). Special IssueCzech J. Food Sci.

[fsn32516-bib-0056] Anderson Shirley , Aldana S , Beggs M , Birkey J , Conquest A , Conway R , Hemminger T , Herrick J , Hurley C , Ionita C , Longbind J , McMaignal S , Milu A , Mitchell T , Nanke K , Perez A , Phelps M , Reitz J , Salazar A , … Zerr S (2007). Determination of fat, moisture, and protein in meat and meat products by using the FOSS FoodScan Near‐Infrared Spectrophotometer with FOSS Artificial Neural Network Calibration Model and Associated Database: Collaborative study. Journal of AOAC International, 90, (4), 1073–1083. 10.1093/jaoac/90.4.1073 17760345

[fsn32516-bib-0005] AOAC . (1995). Official methods of analysis. Association of Official Analytical Chemists.

[fsn32516-bib-0006] Aussanasuwannakul, A. , Brett Kenney, P. , Weber, G. M. , Yao, J. , Slider, S. D. , Manor, M. L. , & Salem, M. (2011). Effect of sexual maturation on growth, fillet composition, and texture of female rainbow trout (*Oncorhynchus mykiss*) on a high nutritional plane. Aquaculture, 317(1–4), 79–88. 10.1016/j.aquaculture.2011.04.015

[fsn32516-bib-0007] Caballero, M. J. , Betancor, M. , Escrig, J. C. , Montero, D. , De Los Monteros, A. E. , Castro, P. , & Izquierdo, M. (2009). Post mortem changes produced in the muscle of sea bream (*Sparus aurata*) during ice storage. Aquaculture, 291(3–4), 210–216. 10.1016/j.aquaculture.2009.03.032

[fsn32516-bib-0008] Castro, P. L. , Caballero, M. J. , Ginés, R. , Penedo, J. C. , Montero, D. , Lastilla, M. T. , & Izquierdo, M. (2015). Linseed oil inclusion in sea bream diets: Effect on muscle quality and shelf life. Aquaculture Research, 46(1), 75–85. 10.1111/are.12161

[fsn32516-bib-0009] Castro, P. L. , Caballero, M. J. , Millán, R. , Ginés, R. , Montero, D. , & Izquierdo, M. (2010). Linseed oil inclusion in sea bream diets: Effect on fatty acid composition during ice storage. European Journal of Lipid Science and Technology, 112(9), 985–993. 10.1002/ejlt.200900240

[fsn32516-bib-0010] Castro, P. L. , Rincón, L. , Álvarez, B. , Rey, E. , & Ginés, R. (2018). Blackspot seabream (*Pagellus bogaraveo*) fed different diets. Histologic study of the lipid muscle fiber distribution and effect on quality during shelf life. Aquaculture, 484, 71–81. 10.1016/j.aquaculture.2017.10.042

[fsn32516-bib-0011] Cheng, J. H. , Sun, D. W. , Han, Z. , & Zeng, X. A. (2014). Texture and structure measurements and analyses for evaluation of fish and fillet freshness quality: A review. Comprehensive Reviews in Food Science and Food Safety, 13(1), 52–61. 10.1111/1541-4337.12043 33412693

[fsn32516-bib-0012] FAO . (2021). Food and Agricultural Organization of the United Nations. http://www.org/fishery/statistics/global‐capture‐production/

[fsn32516-bib-0013] Figueiredo‐Silva, A. C. , Corraze, G. , Borges, P. , & Valente, L. M. (2010). Dietary protein/lipid level and protein source effects on growth, tissue composition and lipid metabolism of blackspot seabream (*Pagellus bogaraveo*). Aquaculture Nutrition, 16(2), 173–187. 10.1111/j.1365-2095.2009.00649.x

[fsn32516-bib-0014] Figueiredo‐Silva, A. C. , Corraze, G. , Rema, P. , Sanchez‐Gurmaches, J. , Gutiérrez, J. , & Valente, L. M. (2009). Blackspot seabream (*Pagellus bogaraveo*) lipogenic and glycolytic pathways appear to be more related to dietary protein level than dietary starch type. Aquaculture, 291(1–2), 101–110. 10.1016/j.aquaculture.2009.03.003

[fsn32516-bib-0015] Francis, D. S. , & Turchini, G. M. (2017). Retro‐engineering the protein sparing effect to preserve n‐3 LC‐PUFA from catabolism and optimise fish oil utilisation: A preliminary case study on juvenile Atlantic salmon. Aquaculture, 468, 184–192. 10.1016/j.aquaculture.2016.10.013

[fsn32516-bib-0016] Fuentes, A. , Fernández‐Segovia, I. , Serra, J. A. , & Barat, J. M. (2010). Comparison of wild and cultured sea bass (*Dicentrarchus labrax*) quality. Food Chemistry, 119(4), 1514–1518. 10.1016/j.foodchem.2009.09.036

[fsn32516-bib-0017] García De La Serrana, D. , Fontanillas, R. , Koppe, W. , Fernández‐Borrás, J. , Blasco, J. , & Gutiérrez, J. (2013). Effects of variable protein and lipid proportion in gilthead sea bream (*Sparus aurata*) diets on fillet structure and quality. Aquaculture Nutrition, 19(3), 368–381. 10.1111/j.1365-2095.2012.00966.x

[fsn32516-bib-0018] Ginés, R. , Palicio, M. , Zamorano, M. J. , Argüello, A. , López, J. L. , & Afonso, J. M. (2002). Starvation before slaughtering as a tool to keep freshness attributes in gilthead sea bream (*Sparus aurata*). Aquaculture International, 10(5), 379–389. 10.1023/A:1023365025292

[fsn32516-bib-0019] Ginés, R. , Valdimarsdottir, T. , Sveinsdottir, K. , & Thorarensen, H. (2004). Effects of rearing temperature and strain on sensory characteristics, texture, colour and fat of Arctic charr (*Salvelinus alpinus*). Food Quality and Preference, 15(2), 177–185. 10.1016/S0950-3293(03)00056-9

[fsn32516-bib-0020] Grigorakis, K. , Alexis, M. N. , Taylor, K. D. A. , & Hole, M. (2002). Comparison of wild and cultured gilthead sea bream (*Sparus aurata*); composition, appearance and seasonal variations. International Journal of Food Science & Technology, 37(5), 477–484. 10.1046/j.1365-2621.2002.00604.x

[fsn32516-bib-0021] Haugen, T. , Kiessling, A. , Olsen, R. E. , Rørå, M. B. , Slinde, E. , & Nortvedt, R. (2006). Seasonal variations in muscle growth dynamics and selected quality attributes in Atlantic halibut (*Hippoglossus hippoglossus* L.) fed dietary lipids containing soybean and/or herring oil under different rearing regimes. Aquaculture, 261, 565–579. 10.1016/j.aquaculture.2006.08.012

[fsn32516-bib-0022] Hyldig, G. , & Nielsen, D. (2001). A review of sensory and instrumental methods used to evaluate the texture of fish muscle. Journal of Texture Studies, 32(3), 219–242. 10.1111/j.1745-4603.2001.tb01045.x

[fsn32516-bib-0023] Ingebrigtsen, I. A. , Berge, G. M. , Ruyter, B. , Kjær, M. A. , Mørkøre, T. , Sørensen, M. , & Gjøen, T. (2014). Growth and quality of Atlantic cod (*Gadus morhua*) fed with high and low fat diets supplemented with glutamate. Aquaculture, 433, 367–376. 10.1016/j.aquaculture.2014.06.036

[fsn32516-bib-0024] Izquierdo, M. S. , Obach, A. , Arantzamendi, L. , Montero, D. , & Robaina, L. (2003). Dietary lipid sources for sea bream and seabass: Growth performance, tissue composition and flesh quality. Aquaculture Nutrition, 9(6), 397–407. 10.1046/j.1365-2095.2003.00270.x

[fsn32516-bib-0025] Johnston, I. A. (1999). Muscle development and growth: Potential implications for flesh quality in fish. Aquaculture, 177, 99–115. 10.1016/S0044-8486(99)00072-1

[fsn32516-bib-0026] Johnston, I. A. (2001). Genetic and environmental determinants of muscle growth patterns. W. Hoar & A. Farrell Fish physiology: Muscle development and growth (vol. 18, pp. 141‐186). Gulf Professional Publishing.

[fsn32516-bib-0027] Johnston, I. A. , Li, X. , Vieira, V. L. A. , Nickell, D. , Dingwall, A. , Alderson, R. , Campbell, P. , & Bickerdike, R. (2006). Muscle and flesh quality traits in wild and farmed Atlantic salmon. Aquaculture, 256, 323–336. 10.1016/j.aquaculture.2006.02.048

[fsn32516-bib-0028] Johnston, I. A. , Manthri, S. , Smart, A. , Campbell, P. , Nickell, D. , & Alderson, R. (2003). Plasticity of muscle fibre number in seawater stages of Atlantic salmon in response to photoperiod manipulation. The Journal of Experimental Biology, 206, 3425–3435. 10.1242/jeb.00577 12939373

[fsn32516-bib-0029] Laconisi, V. , Marono, S. , Parisi, G. , Gasco, L. , Genovese, L. , Maricchiolo, G. , & Piccolo, G. (2017). Dietary inclusion of Tenebrio molitor larvae meal: Effects on growth performance and final quality treats of blackspot sea bream (Pagellus bogaraveo). Aquaculture, 476, 49–58. 10.1016/j.aquaculture.2017.04.007

[fsn32516-bib-0030] Lefevre, F. , Cardinal, M. , Bugeon, J. , Labbe, L. , Medale, F. , & Quillet, E. (2015). Selection for muscle fat content and triploidy affect flesh quality in pan‐size rainbow trout, *Oncorhynchus mykiss* . Aquaculture, 448, 569–577. 10.1016/j.aquaculture.2015.06.029

[fsn32516-bib-0031] Li, X. M. , Yuan, J. M. , Fu, S. J. , & Zhang, Y. G. (2016). The effect of sustained swimming exercise on the growth performance, muscle cellularity and flesh quality of juvenile qingbo (*Spinibarbus sinensis*). Aquaculture, 465, 287–295. 10.1016/j.aquaculture.2016.09.021

[fsn32516-bib-0032] Lie, O. (2001). Flesh quality – the role of nutrition. Aquaculture Research, 32(Suppl. 1), 341–348. 10.1046/j.1355-557x.2001.00026.x

[fsn32516-bib-0033] Lorance, P. (2011). History and dynamics of the overexploitation of the blackspot sea bream (*Pagellus bogaraveo*) in the Bay of Biscay. ICES Journal of Marine Science, 68(2), 290–301. 10.1093/icesjms/fsq072

[fsn32516-bib-0034] Luna, L. G. (1968). Manual of histologic staining methods of the armed forces institute of pathology (3rd ed. pp. 168–169). McGraw Hill Book Company.

[fsn32516-bib-0035] Másílko, J. , Zajíc, T. , & Hlavác, D. (2015). The culture system affects organoleptic properties and lipid composition of common carp (*Cyprinus carpio* L.) meat. Journal of Texture Stududies, 46, 345–352. 10.1111/jtxs.12134

[fsn32516-bib-0036] Matos, E. , Gonçalves, A. , Bandarra, N. , Colen, R. , Nunes, M. L. , Valente, L. M. P. , & Dias, J. (2012). Plant proteins and vegetable oil do not have detrimental effects on post‐mortem muscle instrumental texture, sensory properties and nutritional value of gilthead seabream. Aquaculture, 358–359, 205–212. 10.1016/j.aquaculture.2012.07.009

[fsn32516-bib-0037] Menoyo, D. , Izquierdo, M. S. , Robaina, L. , Ginés, R. , Lopez‐Bote, C. J. , & Bautista, J. M. (2004). Adaptation of lipid metabolism, tissue composition and flesh quality in gilthead sea bream (*Sparus aurata*) to the replacement of dietary fish oil by linseed and soyabean oils. British Journal of Nutrition, 92(1), 41–52. 10.1079/BJN20041165 15230986

[fsn32516-bib-0038] Olsson, G. B. , Olsen, R. L. , Carlehög, M. , & Ofstad, R. (2003). Seasonal variations in chemical and sensory characteristics of farmed and wild Atlantic halibut (*Hippoglossus hippoglossus*). Aquaculture, 217(1–4), 191–205. 10.1016/S0044-8486(02)00191-6

[fsn32516-bib-0039] Palstra, A. P. , & Planas, J. V. (2011). Fish under exercise. Fish Physiology and Biochemistry, 37, 259–272. 10.1007/s10695-011-9505-0 21611721 PMC3107430

[fsn32516-bib-0040] Periago, Ma J , Ayala, Ma D , López‐Albors, O. , Abdel, I. , Martínez, C. , García‐Alcázar, A. , Ros, G. , & Gil, F. (2005). Muscle cellularity and flesh quality of wild and farmed sea bass, *Dicentrarchus labrax* L. Aquaculture, 249, 175–188. 10.1016/j.aquaculture.2005.02.047

[fsn32516-bib-0041] Pinho, M. , Diogo, H. , Carvalho, J. , & Gil‐Pereira, J. (2014). Harvesting juveniles of blackspot sea bream (*Pagellus bogaraveo*) in the Azores (Northeast Atlantic): Biological implications, management, and life cycle considerations. ICES Journal of Marine Science, 71(9), 2448–2456. 10.1093/icesjms/fsu089

[fsn32516-bib-0042] Rasmussen, R. S. , López‐Albors, O. , & Alfnes, F. (2013). Exercise effects on fish quality and implications for consumer preferences. In A. P. Palstra , & J. V. Planas (Eds.), Swimming physiology of fish (pp. 275–300). Spriger‐Verlag.

[fsn32516-bib-0043] Rincón, L. , Castro, P. L. , Álvarez, B. , Hernández, M. D. , Álvarez, A. , Claret, A. , Guerrero, L. , & Ginés, R. (2016). Differences in proximal and fatty acid profiles, sensory characteristics, texture, colour and muscle cellularity between wild and farmed blackspot seabream (*Pagellus bogaraveo*). Aquaculture, 451, 195–204. 10.1016/j.aquaculture.2015.09.016

[fsn32516-bib-0044] Sant’Ana , L. S. , Soares , S. , & Vaz‐Pires , P. (2011). Development of a quality index method (QIM) sensory scheme and study of shelf‐life of ice‐stored blackspot seabream (Pagellus bogaraveo). LWT‐Food Science and Technology, 44(10), 2253–2259. 10.1016/j.lwt.2011.07.004

[fsn32516-bib-0045] Silva, P. , Andrade, C. A. P. , Timoteo, V. M. F. A. , Rocha, E. , & Valente, L. M. P. (2006). Dietary protein, growth, nutrient utilization and body composition of juvenile blackspot seabream, *Pagellus bogaraveo* (Brunnich). Aquaculture Research, 37(10), 1007–1014. 10.1111/j.1365-2109.2006.01520.x

[fsn32516-bib-0046] Silva, P. , Rowlerson, A. M. , Valente, L. M. P. , Olmedo, M. , Monteiro, R. A. F. , & Rocha, E. (2008). Muscle differentiation in blackspot seabream (*Pagellus bogaraveo*, Brunnich): Histochemical and immunohistochemical study of the fibre types. Tissue and Cell, 40(6), 447–458. 10.1016/j.tice.2008.05.001 18620718

[fsn32516-bib-0047] Silva, P. , Valente, L. M. P. , Olmedo, M. , Galante, M. H. , Monteiro, R. A. F. , & Rocha, A. E. (2009). Hyperplastic and hypertrophic growth of lateral muscle in blackspot seabream (*Pagellus bogaraveo*) from hatching to juvenile. Journal of Fish Biology, 74, 37–53. 10.1111/j.1095-8649.2008.02122.x 20735523

[fsn32516-bib-0048] Suárez, M. D. , Martínez, T. F. , Sáez, M. I. , Morales, A. E. , & García‐Gallego, M. (2010). Effects of dietary restriction on post‐mortem changes in white muscle of sea bream (*Sparus aurata*). Aquaculture, 307(1–2), 49–55. 10.1016/j.aquaculture.2010.07.006

[fsn32516-bib-0049] Tacon, A. G. J. , & Metian, M. (2008). Global overview on the use of fish meal and fish oil in industrially compounded aquafeeds: Trends and future prospects. Aquaculture, 285, 146–158. 10.1016/j.aquaculture.2008.08.015

[fsn32516-bib-0050] Thakur, D. P. , Morioka, K. , Itoh, N. , Wada, M. , & Itoh, Y. (2009). Muscle biochemical constituents of cultured amberjack *Seriola dumerili* and their influence on raw meat texture. Fisheries Science, 75, 1489–1498. 10.1007/s12562-009-0173-2

[fsn32516-bib-0051] Thakur, D. P. , Morioka, K. , Itoh, Y. , & Obatake, A. (2003). Lipid composition and deposition of cultured yellowtail *Seriola quinqueradiata* muscle at different anatomical locations in relation to meat texture. Fisheries Science, 69(3), 487–494. 10.1046/j.1444-2906.2003.00649.x

[fsn32516-bib-0052] Valente, L. M. P. , Linares, F. , Villanueva, J. L. R. , Silva, J. M. G. , Espe, M. , Escórcio, C. , & Peleteiro, J. B. (2011). Dietary protein source or energy levels have no major impact on growth performance, nutrient utilisation or flesh fatty acids composition of market‐sized Senegalese sole. Aquaculture, 318(1–2), 128–137. 10.1016/j.aquaculture.2011.05.026

[fsn32516-bib-0053] Valente, L. M. P. , Olmedo, M. , Borges, P. , Soares, S. , Gomes, E. F. S. , Álvarez‐Blázquez, B. , & Linares, F. (2010). Effects of carbohydrate sources on growth, body composition and tissue lipid deposition of blackspot seabream, *Pagellus bogaraveo* (Brunnich). Journal of Animal Physiology and Animal Nutrition, 94(2), 212–219. 10.1111/j.1439-0396.2008.00900.x 19175453

[fsn32516-bib-0054] Veland, J. O. , & Torrissen, M. O. J. (1999). The texture of Atlantic salmon (*Salmo salar*) muscle as measured instrumentally using TPA and Warner‐Brazler shear test. Journal of the Science of Food and Agriculture, 79, 1737–1746. 10.1002/(SICI)1097-0010(199909)79:12<1737:AID-JSFA432>3.0.CO;2-Y

[fsn32516-bib-0055] Xu, H. , Dong, X. , Zuo, R. , Mai, K. , & Ai, Q. (2016). Response of juvenile Japanese seabass (*Lateolabrax japonicus*) to different dietary fatty acid profiles: Growth performance, tissue lipid accumulation, liver histology and flesh texture. Aquaculture, 461, 40–47. 10.1016/j.aquaculture.2016.04.023

